# REM Sleep Fragmentation in Patients With Short-Term Insomnia Is Associated With Higher BDI Scores

**DOI:** 10.3389/fpsyt.2021.733998

**Published:** 2021-09-09

**Authors:** Danjuan Wu, Maoqing Tong, Yunxin Ji, Liemin Ruan, Zhongze Lou, He Gao, Qing Yang

**Affiliations:** ^1^Sleep Disorder Diagnosis and Treatment Center, Department of Psychiatric, Ningbo City First Hospital, Ningbo Hospital of Zhejiang University, Zhejiang, China; ^2^Central Laboratory of the Medical Research Center, Ningbo City First Hospital, Ningbo Hospital of Zhejiang University, Zhejiang, China; ^3^Department of Psychiatric, Ningbo Kangning Hospital, Zhejiang, China

**Keywords:** polysomnography, REM fragmentation, acute insomnia, sleep arousal, depression

## Abstract

**Objective:** To observe the changes in sleep characteristics and BDI scores in patients with short-term insomnia disorder (SID) using a longitudinal observational study.

**Methods:** Fifty-four patients who met the criteria for SID of the International Classification of Sleep Disorders, third edition, were recruited. Depression levels were assessed using the Beck depression inventory (BDI) at enrollment and after 3 months of follow-up, respectively. Sleep characteristics were assessed by polysomnography.

**Results:** After 3 months of follow-up, the group was divided into SID with increased BDI score (BDI >15) and SID with normal BDI score (BDI ≤ 15) according to the total BDI score of the second assessment. The differences in rapid eye movement (REM) sleep latency, REM sleep arousal index, and NREM sleep arousal index between the two groups were statistically significant. The total BDI score was positively correlated with REM and NREM sleep arousal index and negatively correlated with REM sleep latency, which were analyzed by Pearson correlation coefficient. Multiple linear regression was used to construct a regression model to predict the risk of depression in which the prediction accuracy reached 83.7%.

**Conclusion:** REM sleep fragmentation is closely associated with future depressive status in patients with SID and is expected to become an index of estimating depression risk.

## Background

Insomnia disorder is the most common sleep disorder worldwide. It is an important public health problem not only because of its high rate of prevalence and disability, but also because insomnia has been found to be an important risk factor for various psychiatric disorders (1–3), especially major depression disorder. Studies show that people with insomnia are two to three times more likely to develop depression than those without insomnia (4, 5), and other researchers believe that the core problem of insomnia involves an overactive arousal system. During sleep, frequent arousals can lead to unstable or fragmented sleep architecture (6–8). Because *rapid eye movement (REM)* sleep represents a state of high brain arousal during sleep, it plays a key role in insomnia (9–11). At the same time, REM sleep is shown to play an important role in the reprocessing and consolidation of emotional experiences in the limbic system (12). A previous study (13) finds that changes in REM sleep are frequently observed in patients with major depression, for example, shorter REM sleep latency, increased REM sleep duration, and REM density. Excessive arousal and REM sleep fragmentation play an important role in the mood regulation of insomnia and depression by affecting the elimination of negative emotions. The bidirectional relationship between these two disorders may also suggest that instability of REM sleep has a particular impact on insomnia and depression and may be a key factor in the common mechanism of action of insomnia and depression (11, 14). The concept of instability of REM sleep is based on evidence showing increased micro- and macro-arousals during REM sleep in insomnia patients (15). REM sleep is involved in processes that affect emotional homeostasis in the brain. Prolonged and sustained disturbance of REM sleep through arousal or wake intrusion leads to decreased REM stability during sleep (16). REM sleep fragmentation is one of the pathological bases of depression in patients with chronic insomnia (17, 18). Therefore, stability of REM sleep is important for insomnia patients.

In our study, polysomnography (PSG) was used to analyze and assess the sleep architecture of patients with short-term insomnia, to explore the effects of REM sleep segments and REM-related sleep architecture on the mood of patients with short-term insomnia disorder (SID), and to try to construct regression models that can better predict the risk of depression.

## Materials and Methods

### Patients

Patients attended the sleep clinic and psychological consultation clinic of Ningbo First Hospital from January 2019 to September 2020. They met the diagnostic criteria for SID of the International Classification of Sleep Disorders, third edition (19): age >18 years, male and female, total duration of illness <3 months, able to cooperate with the completion of questionnaires and PSG monitoring, and could comply with study requirements for follow-up. Exclusion criteria were untreated physical disease; a previous history of epilepsy, depressive disorder, schizophrenia, bipolar disorder, anxiety disorder, neurodevelopmental delay, or cognitive disorder; shift workers; travel across three or more time zones within 14 days prior to enrollment; a previous history of other sleep disorders or PSG monitoring revealing the following conditions: sleep apnea hypopnea syndrome, apnea hypopnea index (AHI) >15 breaths/h, REM AHI (RAHI) >5 times/h; oxygen saturation <90%; periodic leg movement syndrome: periodic leg movement index (PLMI) >5 times/h; heterogeneous sleep. All subjects did not receive any psychological, physical, and/or pharmacological treatment between 7 days prior to admission and 3 months of follow-up.

### Methods

#### Phase I: Clinical Interview, Psychological Assessment, and Baseline Data Collection

We recorded baseline data on subjects' age, sex, body mass index, smoking, and alcohol consumption by telephone and clinic visits. After subjects were enrolled, psychological evaluations included the Pittsburgh Sleep Quality Index (PSQI) (20), a 19-item questionnaire that assesses sleep quality and disorders over a 1-month interval. The first four items are open-ended questions, and items 5–19 are scored using a 4-point Likert scale. Scores for the seven components were obtained, and the scores were summed to obtain a total score ranging from 0 to 21. A score >5 indicates poor sleep quality. The Beck Anxiety Inventory (BAI) (21), developed by 21, contains 21 self-administered anxiety questionnaires that reflect the severity of anxiety, and a BAI ≥45 is used as a criterion for positive anxiety. The Beck Depression Inventory (BDI) (22) was developed by 22. The whole scale is divided into 21 groups, which are divided into 0–3 levels. After completing the sum of the parts, the total score was divided into four levels with total scores as follows: ≤ 10: healthy and no depression, 10–15: bad mood and attention, and 15–25: depression. A total score >25 indicates that depression is severe, and the patient must be seen by a psychiatrist. Subjects were followed up for 3 months, and then depression was assessed again using the BDI.

A trained psychometrician administered the questionnaire to the patients, informing them of the purpose of the study, the protocol, and precautions. The subject gave informed understanding and consent and then began to fill out the questionnaire. After completion of the questionnaire, the participant returned it, and the staff checked the completeness of the content.

#### Phase II: Sleep Laboratory Tests

Subjects who met the enrollment criteria underwent PSG within 1 week, and patients were given a sleep diary record to determine their resting patterns while waiting for PSG. The specific information of the polysomnograph used in the study is as follows: model and specification: Grael, medical device registration certificate number: 20172210823, manufacturer: Compumedics Limited. Sleep lab tests include one night of PSG monitoring. The PSG monitoring consists mainly of six leads of EEG activity (EEG: F3-M2, F4-M1, C3-M2, C4-M1, O1-M2, O2-M1), electromyographic activity of the jaw and tibialis anterior muscles (EMG), bilateral electro-ocular activity (EOG), electrocardiogram (ECG), oral and nasal airflow, chest and abdominal movements, oxygen saturation, and snoring. The measured parameters include stage REM sleep latency (RL): sleep onset to first epoch of Stage REM in minutes; number of REM sleep cycles (RC); total sleep time (TST) in minutes; REM sleep arousal index (REM-ArI): number of arousals in stage REM × 60/TST; NREM sleep arousal index (NREM-ArI): number of arousals in stage NREM × 60/TST; REM sleep duration (RD): time in stage REM; percentage of TST in stage REM (REM%): (time in stage REM/TST) × 100; percentage of TST in stage NREM (NREM%): (time in stage NREM/TST) × 100; and sleep efficiency (SE): (TST/time in bed) × 100. The installation of the PSG equipment and the analysis of the PSG were performed by professional PSG technicians under the technical guidance of a physician licensed as a registered PSG technician in the United States. The rules, terminology, and technical specifications of the American Association for Sleep Research Manual of Interpretation of Sleep and Associated Events version 2.6 (AASM) were used (23) as the interpretation criteria to determine sleep staging and sleep events.

### Statistical Methods

Statistical analysis was performed using SPSS 26.0. Measured data were expressed as mean ± standard deviation (x ± s), count data were expressed as percentages, mean values between groups were compared using independent samples *t*-test and chi-square (χ^2^) test, and Levene's homogeneity of variance test was used for independent samples *t*-test to test the difference in *t*-distribution of each index between two groups. If *P* > 0.05, there is a statistical difference. Both *t*-test and chi-square test (χ^2^) were used as two-sided tests, and *P* < 0.05 was considered statistically significant. Pearson analysis was performed to analyze the correlation between multiple variables. Regression models were created using multiple linear regression, and their correlation was assessed using covariance diagnostics in linear regressions between multiple variables to determine whether these variables could be modeled. The Durbin-Watson (D-W) test was used to determine whether the independence conditions for linear regression were met. Analysis of variance was used to determine that the constructed regression model was statistically significant in the range of *P* < 0.05. Regression coefficients were used to indicate the degree of influence between the four constants of the REM arousal index, the number of REM cycles, REM latency, the number of REM arousals, and the dependent variable of depression score, and the regression coefficient *P* < 0.05 was statistically significant.

## Results

### Overall

A total of 54 patients with SID were included, 28 (52%) of them were male and 26 (48%) were female. The mean age was (45.20 ± 13.92) years, and body mass index was (22.67 ± 2.73). The BDI score at admission was (6.17 ± 3.73). After 3 months of follow-up, BDI score was (10.07 ± 7.66); insomnia symptoms were relieved in 36 subjects and persisted in 18 subjects. Eleven of these patients (20%) had a BDI score >15, which implies 20% risk of depression in patients with SID after 3 months of follow-up. In the group with persistent insomnia symptoms, the probability of having a BDI score >15 was 50%, whereas in the group with recovery from SID, the probability of having a BID score >15 was only 6%. We define recovery from SID as subjects having 7 or more weeks of good sleep after an episode of SID at a 3-month follow-up, of which the last 4 weeks must be designated as good sleep. Good sleep: five or more nights per week requiring a sleep latency of 30 min or less with 5% TST or less time awake after sleep during the night. Total sleep duration is 6–10 h without daytime symptoms (19, 24).

### Baseline Characteristics

After 3 months of follow-up, the group was divided into SID with increased BDI score (BDI >15) and SID with normal BDI score (BDI ≤ 15) groups according to the BDI score of the second assessment. There was no statistical difference in baseline levels between the two groups except for age differences (Table 1).

**Table 1:** Baseline characteristics of 54 SID patients (BDI score classification after 3 months of follow-up).

**Baseline data**	BDI score^*^ ≤15	BDI score^*^ >15	**χ^**2**^ or**	***p*-value**
	**(*n* = 43)**	**(*n* = 11)**	***T*-value**	
	**Mean or %**	**Mean or %**		
Age	47.23 ± 12.71	37.27 ± 16.17	2.192	0.033
Female (%)	20 (47%)	6 (55%)	0.226	0.634
Smoking history (%)	4 (9%)	3 (27%)	2.507	0.113
Drinking history (%)	5 (12%)	3 (27%)	1.699	0.192
BMI(kg/cm^2^)	22.63 ± 2.78	22.82 ± 2.6	−0.202	0.841
BDI score at the time of enrollment	5.98 ± 3.93	6.91 ± 2.84	−0.737	0.465
BAI score	10.33 ± 5.64	10.55 ± 7.09	−0.109	0.913
PLMI (times/hour)	0.27 ± 0.93	0.14 ± 0.45	0.474	0.372
AHI (times/hour)	2.60 ± 4.12	1.48 ± 3.41	0.824	0.413
REM-AHI (times/hour)	1.02 ± 1.47	0.53 ± 1.18	1.022	0.311
NREM-AHI (times/hour)	2.89 ± 4.72	1.67 ± 3.81	0.788	0.434
PSQI score	12.79 ± 3.91	15.91 ± 3.70	−2.383	0.809

*BDI score*

* *: BDI score at the second assessment after 3 months of follow-up. BMI, Body Mass Index; BDI, Beck Depression Inventory; BAI, Beck Anxiety Inventory; PLMI, periodic limb movement index; AHI, Sleep apnea index; PSQI, Pittsburgh Sleep Quality Index; REM, rapid eye moment; NRME, non-rapid eye moment*.

### Sleep Characteristics

The statistical method of independent samples *t*-test was used to compare the differences between the two groups of indicators (Table 2): the REM sleep latency (*t* = 4.877, *P* = 0.003), the REM sleep arousal index (*t* = −3.627, *P* = 0.004) and the NREM sleep arousal index (*t* = −2.662, *P* = 0.010) with statistically significant differences.

**Table 2:** Independent sample *t*-test of sleep structure of the two groups of patients (BDI score classification after 3 months of follow-up).

**Sleep structure**	**BDI score** **≤15 (*n* = 43)**	**BDI score** **>15 (*n* = 11)**	***F*-value**	***T*-value**	***P*-value**
RL (min)	167.64 ± 80.85	86.15 ± 33.40	13.399	4.877	0.003
RCs	2.79 ± 1.57	2.18 ± 1.08	3.174	1.213	0.231
TST (min)	370.32 ± 114.81	397.14 ± 102.11	0.187	−0.706	0.484
REM-ArI	4.13 ± 2.04	11.01 ± 6.21	15.948	−3.627	0.004
NREM-ArI	4.25 ± 2.42	6.60 ± 3.32	2.257	−2.662	0.010
RD	51.99 ± 34.80	45.41 ± 28.48	0.305	0.578	0.566
REM (%)	12.78 ± 6.81	11.40 ± 6.37	0.030	0.606	0.547
NREM (%)	86.99 ± 6.83	87.69 ± 6.69	0.006	−0.305	0.762
SE (%)	70.96 ± 17.27	77.44 ± 16.21	0.639	−1.123	0.266

*RL, REM sleep latency; RCs, Number of REM cycles; TST, total sleep time; REM-ArI, REM sleep arousal index; NREM-ArI, NREM sleep arousal index; RD:REM sleep duration; REM (%), REM Sleep time/TST; SE, sleep latency*.

### Relationship Between Sleep Characteristics and Depression

**Figure 1:**
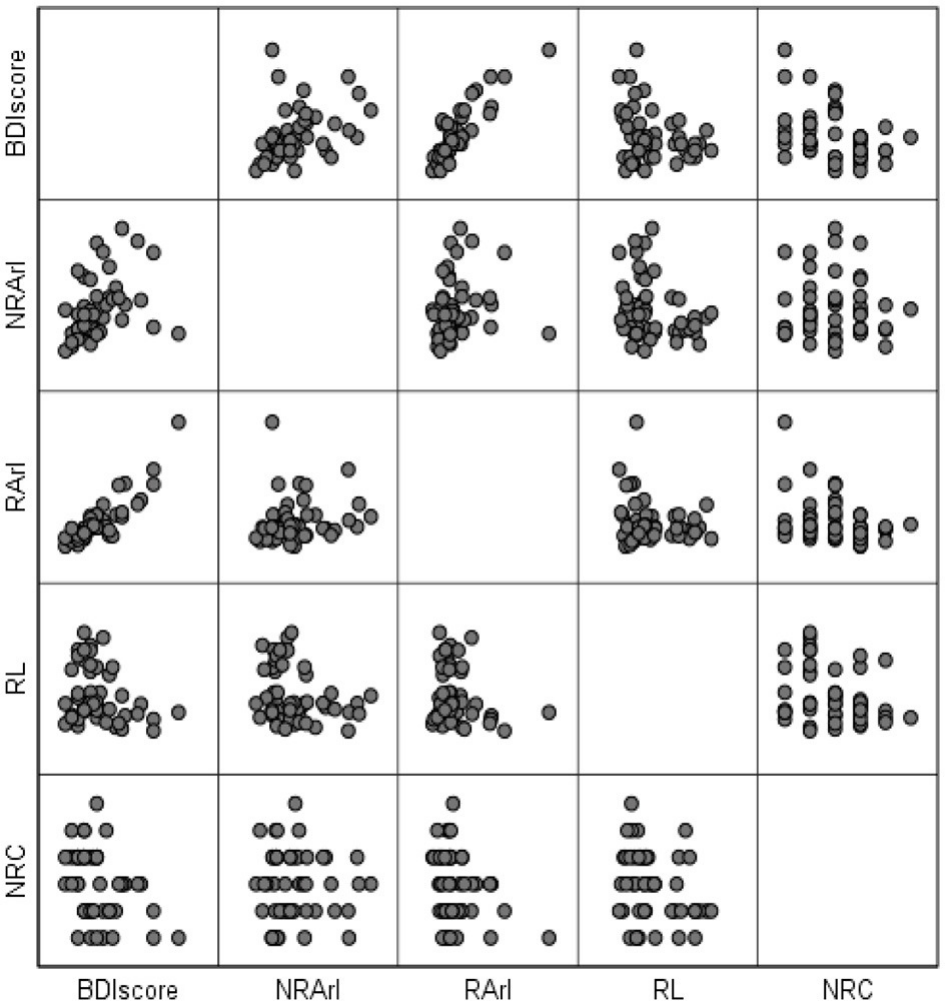
Matrix scatterplot between BDI scores (after 3 months of follow-up) and structural variables of rapid eye movement sleep after 3 months of follow-up. BDI, Beck Depression Inventory; RL, REM sleep latency; REM-ArI, REM sleep arousal index; NREM-ArI, NREM sleep arousal index; RCs, Number of REM cycles.

In linear regression and Pearson correlation analyses (Figure 1; Table 3), it is shown that the BDI score is positively correlated with the REM-ArI and NREM-ArI (significant at the 0.01 level, *P* = 0.000, *P* = 0.002) and negatively correlated with REM sleep latency (significant at the 0.05 level, *P* = 0.046).

**Table 3:** Correlation analysis between the BDI score and various indicators of sleep structure after 3 months of follow-up.

		**BDI (score)**	**RL**	**REM-ArI**	**NREM-ArI**
BDI (score)	Pearson correlation	1	−0.287^*^	0.825^**^	0.416^**^
	Sig. (Two-tailed)		0.046	0.000	0.002
	Number of cases	54	49	54	54
RL	Pearson correlation	−0.287^*^	1	−0.229	−0.285
	Sig. (Two-tailed)	0.046		0.114	0.047
	Number of cases	49	49	49	49
REM-ArI	Pearson correlation	0.825^**^	−0.229	1	0.252
	Sig. (Two-tailed)	0.000	0.114		0.066
	Number of cases	54	49	54	54
NREM-ArI	Pearson correlation	0.416^**^	−0.285	0.252	1
	Sig. (Two-tailed)	0.002	0.047	0.066	
	Number of cases	54	49	54	54

*BDI, Beck Depression Inventory; RL, REM sleep latency; REM-ArI, REM sleep arousal index; NREM-ArI, NREM sleep arousal index*.

* *p < 0.05*,

** *p < 0.01*.

### Regression Prediction Model

We constructed a regression model to further explore the relationship between REM sleep characteristics and BDI scores in patients with SID. Using the diagnosis of covariance in linear regression (Table 4), the correlation between multiple variables in this study was not significant. The D-W test value of 1.924 can be considered to satisfy the independence condition for linear regression. The results of the statistical tests of the model are presented in the analysis of variance (Table 5): the regression model (*F* = 56.564, *p* = 0.000 < 0.05), indicates that the constructed regression model is statistically significant. Therefore, a statistically significant model was constructed in this study, which had a good explanatory power (*R*^2^ = 0.837). The accuracy of the model in predicting the risk of depression was 83.7%. Table 6 shows the coefficients of the regression model used to predict the risk of depression in patients with SID: the standardized coefficient of the REM = ArI was β = 0.772, *p* = 0.000, indicating that the REM-ArI had the greatest effect on the risk of depression across the four variables.

**Table 4:** Summary of regression models for predicting the risk of depression in patients with short-term insomnia.

**Mode**	***R***	***R*^**2**^**	**Adjusted *R*^**2**^**	**Standard estimation error**	**Durbin-Watson (*D-W*)**
	0.915^a^	0.837	0.822	3.21662	1.924

a *Predictors: (constant), RCs, RL, RArI, NRAr*.

**Table 5:** Analysis of variance of the regression model for predicting the risk of depression in patients with short-term insomnia.

**Mode**	**Sum of squares**		**Degree of freedom**	**Mean square**	***F***	***P***
	Regress	2340.993	4	585.248	56.564	0.000^a^
	Residual	455.252	44	10.347		
	Total	2796.245	48			

a *Dependent variable: depression score*.

**Table 6:** Predictive regression model coefficients of depression risk in patients with short-term insomnia.

**Model**	**Unstandardized coefficient**		**Standardization coefficient**	***T***	***P***
	***B***	**Standard error**	**Beta**		
(constant)	2.504	2.646		0.947	0.349
RL	−0.008	0.007	−0.089	−1.261	0.214
REM-ArI	1.438	0.128	0.772	11.206	0.000
NREM-ArI	0.609	0.177	0.220	3.436	0.001
RCs	−0.860	0.418	−0.144	−2.058	0.046

*RL, REM sleep latency; REM-ArI, REM sleep arousal index; NREM-ArI, NREM sleep arousal index; RCs, Number of REM cycles*.

## Discussion

The challenge of the link between insomnia and depression is that insomnia disorder may be both a risk factor for and a consequence of depression. The comorbidity of depression and insomnia disorder did not first attract the attention of researchers, and at first, attention was focused on the latter proposition: that insomnia was a clinical symptom accompanying depressive disorders and that insomnia symptoms would resolve with improvement of depressive symptoms as long as we treated them with antidepressant therapy. Until the early 1990s, several studies showed that preexisting or persistent insomnia symptoms not only failed to resolve with improvement in depressive symptom but instead, increased the risk of depressive episodes and were a risk factor for depressive disorders (25, 26). Therefore, one of our aims in this study was to investigate whether the presence or persistence of insomnia symptoms increases the risk of depressive episodes. By measuring depression in 54 patients with SID using the BDI depression scale, it was found that the probability of having a BDI score >15 was found to be 50% in the group with persistent insomnia symptoms after 3 months of follow-up, and the probability of having a BID score >15 was only 6% in the group with remission of insomnia symptoms. This result suggests that patients with persistent insomnia symptoms are more likely to experience depressive mood. Insomnia is an important marker of depression, which is consistent with the results of many foreign studies (5, 6, 27).

Although we observed that the chronic presence of insomnia symptoms is a risk factor for the development of depression, no studies have been able to explain the specific mechanisms underlying the association between insomnia and depression, and there is a lack of corresponding physiological features or biological characteristics that can predict the risk of depression (28). The quantitative electroencephalogram (EEG) studies show that there are characteristic changes in depression, consisting of disturbed sleep continuity, inductions of REM sleep. Of all the changes, abnormalities in REM sleep are key to the EEG changes in depression (29). It was shown as early as 2009 that REM sleep facilitates the reactivation of previously acquired emotional experiences in the limbic system of the brain and their integration with semantic memory, leading to a decrease in amygdala activity and traces of emotional memory over time (30). This finding suggests that changes in the structure of REM sleep results in blocked reactivation of previously acquired emotional experiences in the limbic system of the brain, leading to diminished amygdala activity and elimination of traces of emotional memory. Studies on how eye movements in REM sleep cause transient, time-specific activation of the amygdala also confirm the role of the limbic system in the reprocessing and consolidation of emotional experiences in REM sleep (12). As observed through several studies (13): changes in the structure of REM sleep, such as shortened REM latency, increased REM sleep duration, and increased density of REM, are frequently observed in people diagnosed with depressive disorders.

Recently, it was found that depression may be related to the dysfunction of a network of structures that regulate REM sleep, such as the limbic system, including the hippocampus, amygdala, and medial pre-frontal cortex. The reward network is dysfunctional and associated with depression symptoms in patients with chronic insomnia disorder (31). Furthermore, the subregions of the medial pre-frontal cortex (mPFC) show great changes in neural activity in depressed patients (32). Anatomic tracing studies show that the mPFC projects to the pontine REM-off neurons in the ventrolateral periaqueductal gray and adjacent lateral pontine tegmentum, which interacts with REM-on neurons in the dorsal pons. Therefore, the ventral mPFC may be a critical area for regulating both depression and sleep, and it is suggested as a critical site for REM sleep abnormalities and other behaviors in depression (33). Depression is reported to be associated with a reduction in 5-hydroxytryptamine neurotransmission, which may explain why depression is characterized by a short REM sleep latency (the 5-hydroxytryptamine system is off-line during REM sleep). That said, the role of 5-hydroxytryptamine neurotransmission between insomnia and depression may be much more complex and may be better characterized by an overall dysregulation of the system (i.e., the system is online when it should be off-line and off-line when it should be online) (28, 34).

Expanding upon traditional ways of looking at REM alterations, recent studies examine REM sleep fragmentation (i.e., the number and duration of short arousals that disrupt the continuity of the REM period). Thus, REM sleep continuity may function to depotentiate emotional load: a function that is disrupted when REM is fragmented by brief arousals (13, 35). The possibility exists that REM sleep fragmentation is linked with less efficient regulation of negative affect. The results of our study found that 54 patients with SID were divided into SID with increased BDI score (BDI >15) and SID with normal BDI score (BDI ≤ 15), and the total BDI score was positively correlated with REM-ArI and NREM-ArI and negatively correlated with REM sleep latency after a 3-month follow-up observation. This structural sleep characteristic could suggest that the presence of shortened REM latency and REM sleep fragmentation (increased microarousal index) in patients with clinical insomnia disorder would increase the risk of depressive episodes. We further developed a regression model to predict the risk of depression to explore the relationship between REM sleep characteristics and depression in patients with SID. The model uses four independent variables, REM-ArI, REM cycle number, REM sleep latency, and NREM-ArI, to predict the risk of subsequent depression with 83.7% accuracy. Further analysis of the regression coefficients revealed that their REM-ArI had the greatest effect on depression. The results suggest that REM sleep fragmentation has an extremely important role in depressive mood changes, there are relatively few studies on specific REM structural characteristics in previous studies, and the study of the relationship between changes in REM structure and depressive mood may further refine the explanation and description of the mechanism that there are many studies reporting that insomnia disorder increases the risk of depressive episodes.

This may also indirectly suggest that insomnia may act on emotion regulation mechanisms by disrupting the structure of REM sleep, thus affecting the reprocessing and consolidation of emotions and leading to a weakening of the effect of eliminating negative emotions. Excessive arousal and REM sleep fragmentation play an important role in emotion regulation in insomnia, depression, and PTSD by affecting the elimination of negative emotions (36). Our study suggests that changes in the structure of REM sleep, especially REM sleep fragmentation, may be a characteristic marker for assessing the risk of depressive episodes and that these patients with short-term insomnia disorders presenting with REM sleep fragmention should be alerted to appropriate interventions to reduce the risk of developing depression.

### Study Limitations

Our study used primarily one night of sleep monitoring data, and therefore, there is some controversy as to whether the results are affected by first-night effects. Although several previous studies show no statistical difference in sleep structure data between subjects on the first and second night (37–39). Another limitation of the study is that in the present study, we mainly assessed depression levels using the BDI scale in patients with SID with a 3-month follow-up without further clarifying the diagnosis according to the diagnostic criteria of depression.

## Data Availability Statement

The raw data supporting the conclusions of this article will be made available by the authors, without undue reservation.

## Ethics Statement

This study was reviewed and approved by the ethics committee of Ningbo First Hospital (No. 2018-R048). The patients/participants provided their written informed consent to participate in this study.

## Author Contributions

DW: conceptualization, methodology, software, data curation, writing-original draft, visualization, investigation, and formal analysis. MT: writing-original draft, writing-review and editing, investigation, and validation. YJ: validation, investigation, and supervision. LR: project administration and validation. ZL: data curation and visualization. HG: investigation and formal analysis. QY: resources and investigation. All authors contributed to the article and approved the submitted version.

## Funding

This work was supported by Medicine and Health Technology Plan Project of Zhejiang Province (Grant No. 2019RC261), Research Fund of traditional Chinese medicine in Zhejiang Province (Grant No. 2018ZB116), Medicine and Health Technology Plan Project of Zhejiang Province (Grant No. 2017KY136), and Major Social Development Special Foundation of Ningbo (Grant No. 2017C510010).

## Conflict of Interest

The authors declare that the research was conducted in the absence of any commercial or financial relationships that could be construed as a potential conflict of interest.

## Publisher's Note

All claims expressed in this article are solely those of the authors and do not necessarily represent those of their affiliated organizations, or those of the publisher, the editors and the reviewers. Any product that may be evaluated in this article, or claim that may be made by its manufacturer, is not guaranteed or endorsed by the publisher.
